# Susceptibility survey of *Ommatissus lybicus* (de Bergevin) populations against deltamethrin and fenitrothion in Oman

**DOI:** 10.1038/s41598-019-48244-8

**Published:** 2019-08-12

**Authors:** Rashad Rasool Khan, Salim Ali Humaid Al-Khatri, Thuwaini Hashil Abdullah Al-Ghafri, Ibtisam Salim Suliman Al-Mazidi, Fatima Gharib Al-Rawahi, Saif Suliman Al-Jabri, Muhammad Hammad Hussain

**Affiliations:** 1 Department of Plant Protection, Directorate General of Agriculture and Livestock Research, Ministry of Agriculture and Fisheries, Muscat, Oman; 2 Department of Entomology, University of Agriculture, Faisalabad, Pakistan; 3 Animal Health Research Center, Directorate General of Agriculture and Livestock Research, Ministry of Agriculture and Fisheries, Muscat, Oman

**Keywords:** Entomology, Biomechanics

## Abstract

Despite of extensive application of insecticides, severe infestation of date palms is reported with *Ommatissus lybicus* every year. The development of insecticide resistance in the field strains receiving heavy pesticide regimes can be a reason for unsuccessful control. Seventeen field strains of *O*. *lybicus* from Oman were appraised for resistance against deltamethrin and fenitrothion in years 2017 and 2018. Eleven field strains depicted susceptibility (RR < 3-fold) while six strains expressed minor (RR = 3–5-fold) to low level (RR = 5–10-fold) of resistance against deltamethrin when compared with lab-susceptible strain. In case of fenitrothion, fourteen field strains exhibited minor to low level of resistance and only two showed susceptibility. Intermediate resistance (RR = 10–40-fold) was also reported in one field strain against fenitrothion. A significant but low resistance (RR = 7.86-fold) was observed in a lab strain selected with deltamethrin for resistance development mechanism studies. The deltamethrin-selected strain exhibited minor resistance development (RR = 3.13-fold) against fenitrothion. Significant reduction in resistance was observed with higher toxicity values when the two pesticides were tested along with piperonyl butoxide (PBO) against all the field collected dubas bug strains. Higher susceptibility of field strains to deltamethrin suggest it a suitable alternate candidate to deter resistance development in *O*. *lybicus*.

## Introduction

The Dubas bug, *Ommatissus lybicus* (de Bergevin) (Homoptera: Fulgoroidea: Tropiduchidae) is a sap sucking insect and is restricted to date palm *Phoenix dactylifera* L., by showing high specificity^1–4^. The pest is recorded and reported from Egypt, Israel, Saudi Arabia, Oman, Iraq, Yemen, United Arab Emirates (UAE), Qatar and Pakistan^5,6^. It completes its two generations (spring and summer) in a year feeding on the leaves^7^ and passes the off-season in egg stage^8^. In Oman, the emergence of nymphs of spring and autumn generations takes place in the months of February and August, respectively^9^. The species has been ranked as major pest of date palm in Oman over the last four decades in terms of its distribution, severity of infestation and consequent losses^10^. Its high infestation destroyed 12 million palms in Morocco during two decades and decreased the production to zero^11^. Moreover, the dense oviposition causes the green plant tissues of the area of eggs insertion to dry up. The larger populations of dubas can destroy the date palms and can cause 25 to 50% crop losses^12^. Its severe infestation caused economic losses up to 50% in Iraq and 28% in Oman^13^. Heavy infestation and intense feeding by the pest weakens the tree, whereas honeydew droplets excreted by the bug cover the leaflets. Further depositions of dust particles and black sooty mould growth reduce the photosynthetic ability of host plants and results in chlorotic appearance of the fronds^14,15^. The northern Oman is declared at high risk and will face severe dubas bug infestations in future due to its climatic suitability^16^.

In order to manage such a notorious pest, the Ministry of Agriculture in Oman has tested several pesticides from pyrethroid and organophosphate groups by aerial and ground applications. The insecticides included dichlorvos, malathion, fenitrothion, etofenprox and combination of fenitrothion and esfenvalerate were used in aerial sprays. In ground application, dichlorvos, deltamethrin, phenthoate and esfenvalerate were used to control dubas bug^13,17–19^.

Many authors reported the toxicities and efficiencies of a number of pesticides including pyrethroids, organophosphates, neonicotinoids and botanicals etc., against *O*. *lybicus* under laboratory and field conditions^20–24^. Insecticides including cypermethrin, deltamethrin, diazinon and fenitrothion were found more toxic and reported to cause high mortality of dubas bug under laboratory and field conditions^25,26^. Cypermethrin and bifenthrin were also reported as more efficient insecticides for dubas bug population reduction up to 35 days of spray^24^. Insecticide injecting in tree trunk (endotherapy) with thiamethoxam and spraying with spinetoram were reported very effective against dubas bug^20,27^. Mineral oil and kaolin were studied as an alternate choice of conventional chemicals and were tested along with diazinon with non-significant results but proved to be equally efficient in field trials^23^.

The indiscriminate ground application and heavy aerial spraying of insecticides can lead to the development of resistance in the target insects. The excessive application of pesticides to date palm orchards to control this pest has contributed to the development of resistance in dubas bug populations to several conventional chemical insecticides^28,29^. Inability to fly over long distances and intensive pesticide exposures of dubas bug populations can result in formation of diverged populations which may result in development of resistant strains^30^. Since, the phenomenon of insecticide resistance development is spatio-temporal^31^, therefore, it is necessary to investigate the resistance status of different dubas bug populations in Oman, receiving intensive sprays of the chemical insecticides. The resistance status of the bug against any insecticide has not yet been reported particularly from the Sultanate of Oman.

Despite of heavy aerial sprays and ground application of insecticides, severe infestation has been reported every year from different governorates of Oman. Assessment of insecticide resistance is very important and can help in devising an effective management strategy against pest insects^32^. Information about the resistance or susceptibility status of dubas bug can aid in applying alternate measures and mitigate the resistance development phenomenon. Therefore, keeping in view the economic importance of dubas bug and lack of knowledge for the resistance in dubas bug against insecticides, the study was designed to determine the resistance level of dubas bug to the most commonly applied insecticides (deltamethrin and fenitrothion). This is the first report of the resistance in dubas bug against the conventional insecticides most commonly used for its control in the Sultanate of Oman. The findings can be utilized as baseline data for future resistance monitoring and to devise a useful strategy for dubas bug management.

## Results

### Response of deltamethrin-selected strain to deltamethrin and fenitrothion

The Lab strain tested previously with deltamethrin (LC_50_ = 0.112; CI = 0.079–0.156 ppm) was selected to further perform the bioassay experiments for resistance development against deltamethrin. The LC_50_ value significantly increased when selection experiments were performed till 7^th^ generation of the selected strain of *O*. *lybicus*. The LC_50_ values were recorded as 0.281, 0.339, 0.375, 0.452, 0.517, 0.541 and 0.765 ppm from G1 to G-7 (Table 1). A significant development of resistance, ranked as low (8.05-fold RR) was observed when the 7^th^ generation (G-7) was exposed to deltamethrin. The RR values significantly increased from 2.96 to 8.05-folds in the 7^th^ generation (Table 1). The last generation (G-7) tested in the selection experiments was further reared up to ten generations without exposure to any insecticide and then it was tested with deltamethrin and fenitrothion to observe the resistance level against the tested insecticides.

**Table 1:** Resistance selection and toxicity of deltamethrin in lab-susceptible strains of *Ommatissus lybicus* under laboratory conditions.

Strain	n^*^	LC_50_^**^(95% CI^***^)	Slope (S.E)	χ^2^	df	P	RR^****^ (95% CI)
G-0 (Lab Susceptible)	150	0.112 (0.079–0.156)	1.41 (0.19)	2.23	3	0.71	1.17 (0.88–1.31)^ns^
G-1	150	0.281 (0.263–0.331)	1.18 (0.27)	1.81	3	0.93	2.96 (2.03–3.71)^+^
G-2	150	0.339 (0.304–0.373)	1.47 (0.31)	1.89	3	0.87	3.57 (2.97–4.11)^+^
G-3	150	0.375 (0.327–0.437)	1.81 (0.27)	2.11	3	0.53	3.95 (3.05–4.76)^+^
G-4	150	0.452 (0.419–0.488)	1.17 (0.21)	2.12	3	0.67	4.76 (3.37–5.29)^+^
G-5	150	0.517 (0.486–0.548)	1.21 (0.19)	2.11	3	0.59	5.44 (4.17–6.23)^+^
G-6	150	0.541 (0.501–0.583)	1.43 (0.23)	2.89	3	0.31	5.69 (4.37–6.99)^+^
G-7	150	0.765 (0.727–0.819)	1.91 (0.27)	2.14	3	0.43	8.05 (5.62–13.47)^+^
G-10	150	0.747 (0.551–1.171)	1.08 (0.15)	2.37	3	0.56	7.86 (4.90–12.66)^+^

*n = number of test insects used in bioassay trials, **LC_50_ = median lethal concentration (ppm), ***CI = confidence interval, ****RR = resistance ratio, calculated by dividing LC_50_ value of a test generation by the corresponding LC_50_ value of the Lab susceptible (Reference) strain, ns = non-significant, and +significantly different from the Lab susceptible (Reference) strain (95% CIs of RR didn’t include 1).

The results revealed that there was a decrease in the resistance against deltamethrin (RR declined from 8.05 to 7.86-fold) but the decrease was not significant (CIs overlap). However, minor resistance was observed when the same generation was tested against fenitrothion (LC_50_ = 0.025; CI = 0.013–0.037 ppm and RR = 3.13-fold).

### Reference line susceptibility to deltamethrin and fenitrothion

The highest susceptibility levels in the bioassay experiments were observed when the laboratory reared susceptible strain (unexposed to any insecticides for the last ten generations) was exposed to the two tested insecticides (deltamethrin and fenitrothion). The 95% confidence interval (CI) values of LC_50_ did not overlap and those of resistance ratios (RRs) did not contain 1, when the results were compared with the tested field strains of *O*. *lybicus*. The median lethal concentration (LC_50_) values for deltamethrin and fenitrothion were 0.095(CI = 0.065–0.123) and 0.008(CI = 0.004–0.013) ppm, respectively (Tables 2 and 3). These values of median lethal concentrations (LC_50_) were used as reference for calculations of resistance ratios (RRs) and evaluation of resistance levels in the tested field strains of *O*. *lybicus*.

**Table 2:** Toxicity of deltamethrin in various field collected strains of *Ommatissus lybicus* under laboratory conditions.

Strain	n^*^	LC_50_^**^(95% CI^***^)	Slope (S.E)	χ^2^	df	P	RR^****^ (95% CI)
**A’Dakhliyah**							
Hidhan (HN)	150	0.127 (0.079–0.181)	1.22 (0.15)	3.01	3	0.29	1.34 (0.93–1.85)^ns^
Wadi Qari (WQ)	150	0.197 (0.149–0.271)	0.97 (0.14)	2.97	3	0.31	2.07 (1.57–2.49)^+^
Al-Faljayn (AFn)	150	0.205 (0.169–0.249)	1.10 (0.16)	4.22	3	0.27	2.15 (1.71–2.53)^+^
Qalat Al-Masalha (QM)	150	0.757 (0.581–1.155)	1.01 (0.14)	2.21	3	0.37	7.97 (4.89–12.61)^+^
Al-Faghrah (AFh)	150	0.213 (0.177–0.254)	1.55 (0.15)	1.61	3	0.49	2.24 (1.57–3.21)^+^
Al-Jailah (AJ)	150	0.122 (0.074–0.173)	2.22 (0.19)	3.22	3	0.21	1.28 (0.91–1.81)^ns^
Nafaa (NA)	150	0.265 (0.155–0.469)	1.64 (0.15)	2.91	3	0.40	2.79 (1.96–3.96)^+^
Al-Uaina (AU)	150	0.309 (0.225–0.443)	1.45 (0.15)	3.19	3	0.35	3.26 (2.27–4.68)^+^
Al-Elayyah (AE)	150	0.137 (0.103–0.174)	2.10 (0.18)	3.20	3	0.23	1.44 (1.03–2.04) ^+^
Al-Kharma (AK)	150	0.300 (0.184–0.444)	1.23 (0.14)	5.11	3	0.11	3.16 (2.16–4.62)^+^
**A’Sharqiyah** (**North**)							
Baa’d (BD)	150	0.535 (0.461–0.612)	1.18 (0.17)	1.61	3	0.41	5.63 (4.81–6.24)^+^
Maqal (ML)	150	0.216 (0.168–0.273)	1.13 (0.14)	0.53	3	0.61	2.27 (1.54–3.35)^+^
Qarr (QR)	150	0.102 (0.078–0.125)	1.52 (0.16)	1.45	3	0.51	1.07 (0.73–1.57)^ns^
Miss (MS)	150	0.424 (0.334–0.571)	1.13 (0.14)	0.08	3	0.71	4.47 (2.98–6.70)^+^
**A’Dhahira**							
An Nujayd (AN)	150	0.237 (0.190–0.295)	1.24 (0.14)	0.62	3	0.71	2.49 (1.71–3.64)^+^
Al-Hajar (AH)	150	0.244 (0.153–0.385)	1.03 (0.14)	3.05	3	0.42	2.57 (1.72–3.83)^+^
**Al-Batinah North**							
Wadi Haibi (WH)	150	0.631 (0.464–0.992)	0.98 (0.14)	0.64	3	0.63	6.64 (4.13–10.71)^+^
DEL-SEL	150	0.747 (0.551–1.171)	1.08 (0.15)	2.37	3	0.56	7.86 (4.90–12.66)^+^
LAB-SUS (ref. Strain)	150	0.095 (0.065–0.123)	1.22 (0.20)	1.05	3	0.81	1.00

^*^n = number of test insects used in bioassay trials, **LC_50_ = median lethal concentration (ppm), ***CI = confidence interval, ****RR = resistance ratio, calculated by dividing LC_50_ value of a field strain by the corresponding LC_50_ value of the Lab susceptible (Reference) strain, ns = non-significant, and +significantly different from the Lab susceptible (Reference) strain (95% CIs of RR didn’t include 1).

**Table 3:** Toxicity of fenitrothion in various field collected strains of *Ommatissus lybicus* under laboratory conditions.

Strain	n^*^	LC_50_^**^(95% CI^***^)	Slope (S.E)	χ^2^	df	P	RR^****^ (95% CI)
**A’Dakhliyah**							
Hidhan (HN)	150	0.030 (0.023–0.040)	1.31 (0.16)	0.89	3	0.86	3.75 (2.01–5.59)^+^
Wadi Qari (WQ)	150	0.091 (0.077–0.112)	1.77 (0.19)	3.91	3	0.14	11.38 (6.74–17.33)^+^
Al-Faljayn (AFn)	150	0.046 (0.037–0.057)	1.57 (0.17)	2.79	3	0.43	5.75 (3.61–9.23)^+^
Qalat Al-Masalha (QM)	150	0.063 (0.050–0.076)	1.89 (0.18)	2.89	3	0.49	7.88 (3.91–13.11)^+^
Al-Faghrah (AFh)	150	0.070 (0.059–0.084)	1.62 (0.15)	2.25	3	0.28	8.75 (4.58–14.04)^+^
Al-Jailah (AJ)	150	0.044 (0.028–0.064)	1.58 (0.15)	5.11	3	0.36	5.50 (2.88–9.77)^+^
Nafaa (NA)	150	0.030 (0.020–0.041)	1.76 (0.17)	3.98	3	0.13	3.75 (1.97–6.68)^+^
Al-Uaina (AU)	150	0.029 (0.015–0.042)	1.77 (0.17)	3.22	3	0.12	3.63 (1.86–6.34)^+^
Al-Elayyah (AE)	150	0.019 (0.010–0.028)	1.89 (0.20)	4.89	3	0.09	2.38 (1.25–4.34)^+^
Al-Kharma (AK)	150	0.025 (0.020–0.030)	1.75 (0.17)	0.65	3	0.84	3.13 (1.63–5.58)^+^
**A’Sharqiyah** (**North**)							
Baa’d (BD)	150	0.065 (0.054–0.078)	1.47 (0.17)	2.18	3	0.29	8.13 (4.47–15.16)^+^
Maqal (ML)	150	0.043 (0.036–0.050)	1.83 (0.16)	0.32	3	0.89	5.38 (2.80–9.38)^+^
Qarr (QR)	150	0.051 (0.041–0.062)	1.31 (0.15)	0.89	3	0.86	6.38 (3.29–11.34)^+^
Miss (MS)	150	0.046 (0.038–0.055)	1.52 (0.15)	2.96	3	0.47	5.75 (2.98–10.12)^+^
**A’Dhahira**							
An Nujayd (AN)	150	0.025 (0.018–0.030)	1.51 (0.16)	2.58	3	0.51	3.13 (1.52–5.40)^+^
Al-Hajar (AH)	150	0.019 (0.003–0.033)	1.86 (0.19)	2.68	3	0.51	2.38 (1.21–4.23)^+^
**Al-Batinah North**							
Wadi Haibi (WH)	150	0.039 (0.016–0.070)	2.03 (0.17)	3.89	3	0.13	4.88 (2.57–8.55)^+^
DEL-SEL	150	0.025 (0.013–0.037)	2.77 (0.25)	3.80	3	0.11	3.13 (1.67–5.50)^+^
LAB-SUS (Ref. Strain)	150	0.008 (0.004–0.013)	1.32 (0.24)	0.48	3	0.87	1.00

*n = number of test insects used in bioassay trials, **LC_50_ = median lethal concentration (ppm), ***CI = confidence interval, ****RR = resistance ratio, calculated by dividing LC_50_ value of a field strain by the corresponding LC_50_ value of the Lab susceptible (Reference) strain, and +significantly different from the Lab susceptible (Reference) strain (95% CIs of RR didn’t include 1).

### Insecticide resistance status

In total, seventeen field strains of *O*. *lybicus* collected from different localities of various governorates of Oman were evaluated for resistance against deltamethrin and fenitrothion from 2017 to 2018. The results displayed in Tables 2 and 3, indicate that no field strain reported high or intermediate levels of resistance against deltamethrin, however, only one field strain (WQ) from A’Dakhliyah governorate exhibited intermediate level of resistance (11.38-fold) with LC_50_ value of 0.091 ppm against fenitrothion. The same population proved to be susceptible (<3.00-fold RR) against deltamethrin with LC_50_ value of 0.197 ppm (Table 2). Three field strains, each from A’Dakhliyah (QM), A’Sharqiyah (North) (BD) and Al-Batinah North (WH) showed low level of resistance to deltamethrin (LC_50_ = 0.535–0.757 ppm; RR = 5.63–7.97-fold). Only three field strains (AU and AK from A’Dakhliyah and MS from A’Sharqiyah (North)) exhibited minor level of resistance (3–5-fold RR) against deltamethrin (Table 2). All the two tested field strains (AN and AH) from A’Dhahira, two strains (ML and QR) from A’Sharqiyah (North) and seven strains (HN, WQ, AFn, AFh, AJ, NA and AE) from A’Dakhliyah governorates were found to be susceptible against deltamethrin (LC_50_ = 0.102–0.265 ppm; RR = 1.07–2.79-fold). The susceptibility of eleven field strains to deltamethrin when compared with the lab strain refers it to be more efficient as compare to fenitrothion.

Fenitrothion proved to be more toxic as compared to deltamethrin when compared on the basis of LC_50_ values of reference as well as field strains. Lowest toxicity of fenitrothion was observed in the field strain (WQ) from A’Dakhliyah governorate with LC_50_ value of 0.091 ppm. Four field strains from A’Sharqiyah (North) (BD, ML, QR and MS) and A’Dakhliyah (AFn, QM, AFh and AJ) governorates exhibited low levels of resistance against fenitrothion (LC_50_ = 0.043–0.070 ppm; RR = 5.38–8.75-fold). Six field strains (HN, NA, AU and AK from A’Dakhliyah; AN from A’Dhahira and WH from Al-Batinah North) displayed minor levels of resistance (3–5-fold RR) against fenitrothion. Only two strains, one from A’Dakhliyah (AE) and one from A’Dhahira governorate (AH) were found susceptible (<3.00-fold RR) against fenitrothion (Table 3).

### Response of different strains to deltamethrin and fenitrothion with PBO

The insecticides (deltamethrin and fenitrothion) were tested along with synergist (PBO) to see the synergistic effects on their toxicities as well as oxidase-based mechanism of resistance. The results of synergism experiments (PBO and insecticides) are listed in Tables 4 and 5. The results revealed significant reduction in the LC_50_ values of the two insecticides when these were applied in the presence of PBO (95% CIs of SRs did not include 1). However, LC_50_ values did not reduced significantly in AFn, AJ and QR strains against deltamethrin and in HN strain against fenitrothion (95% CIs of SRs include 1 and 95% CIs of LC_50_ values overlap) when compared with LC_50_ values of their respective field strain without PBO. The highest SR value (3.09-fold) was calculated in MS strain which was followed by BD (2.47-fold) and QM (2.31-fold) representing the maximum reduction in LC_50_ values and hence increasing the toxicity of deltamethrin (Table 4). Similarly, highest SR value (1.71-fold) was noticed in AFh strain against fenitrothion (Table 5) representing maximum decrease in LC_50_ (0.070 to 0.041ppm). In experiments where deltamethrin was applied along with PBO to the field and deltamethrin-selected (DEL-SEL) strains, highest resistance (RR = 4.51-fold; LC_50_ = 0.411 ppm; 95% CI = 0.364–0.471 ppm) was noticed in the deltamethrin-selected strain, which was reduced from 7.86-fold resistance level. Minor resistance in WH (RR = 3.67-fold) and QM (RR = 3.60-fold) with SR values of 1.89 and 2.31-fold, respectively was determined against deltamethrin. However, all the remaining field strains proved to be susceptible when treated with deltamethrin in the presence of PBO. Three field strains from A’Dakhliyah (WQ, QM, and AFh) and two from A’Sharqiyah (North) (BD and QR) showed low level of resistance when exposed to fenitrothion with PBO (LC_50_ = 0.040–0.058 ppm; RR = 5.71–8.29-fold). Minor resistance (RR = 3–5-fold) against fenitrothion and PBO was observed in HN, AFn, AJ and AU from A’Dakhliyah, ML and MS from A’Sharqiyah (North) and WH from Al-Batinah North governorates. Remaining all field strains (NA, AE, AK, AN and AH) were found to be susceptible when fenitrothion was applied in the presence of PBO. The resistance status of WQ from A’Dakhliyah governorate was changed from intermediate level (11.38-fold) to low level (8.29-fold) against fenitrothion with PBO. Out of eight strains showing low level of resistance (RR = 5–10-fold) against fenitrothion alone, four strains (AFn, AJ, ML and MS) exhibited minor level of resistance (RR = 3–5-fold) when fenitrothion was applied in the presence of PBO.

**Table 4:** Effect of PBO on toxicity of deltamethrin in laboratory and field strains of *Ommatissus lybicus* under laboratory conditions.

Field Strain	n*	LC_50_^**^(95% CI^***^)	Slope (S.E)	χ^2^	df	P	SR^****^(95% CI)	RR^*****^(95% CI)
**A’Dakhliyah**								
Hidhan (HN)	150	0.098 (0.089–0.119)	1.19 (0.20)	1.17	3	0.79	1.29 (1.01–1.63)^+^	1.08 (0.87–1.31)^ns^
Wadi Qari (WQ)	150	0.139 (0.109–0.163)	1.91 (0.18)	3.19	3	0.29	1.42 (1.03–1.87)^+^	1.52 (1.17–1.91)^+^
Al-Faljayn (AFn)	150	0.159 (0.131–0.189)	0.93 (0.14)	2.97	3	0.31	1.29 (0.93–1.71)^ns^	1.75 (1.37–2.19)^+^
Qalat Al-Masalha (QM)	150	0.328 (0.289–0.387)	1.35 (0.15)	3.19	3	0.33	2.31 (2.01–2.73)^+^	3.60 (3.17–4.07)^+^
Al-Faghrah (AFh)	150	0.155 (0.133–0.184)	0.97 (0.14)	2.97	3	0.31	1.37 (1.02–1.99)^+^	1.70 (1.13–2.39)^+^
Al-Jailah (AJ)	150	0.100 (0.087–0.114)	1.51 (0.16)	1.45	3	0.53	1.22 (0.89–1.71)^ns^	1.10 (0.79–1.41)^ns^
Nafaa (NA)	150	0.170 (0.161–0.186)	1.10 (0.16)	3.97	3	0.23	1.56 (1.14–2.07)^+^	1.87 (1.31–2.49)^+^
Al-Uaina (AU)	150	0.186 (0.165–0.207)	1.21 (0.17)	2.97	3	0.31	1.66 (1.21–2.17)^+^	2.04 (1.69–2.41)^+^
Al-Elayyah (AE)	150	0.110 (0.094–0.125)	1.22 (0.15)	2.91	3	0.37	1.24 (1.01–1.51)^+^	1.20 (0.89–1.51)^ns^
Al-Kharma (AK)	150	0.195 (0.167–0.229)	0.99 (0.14)	2.96	3	0.35	1.54 (1.19–1.97)^+^	2.14 (1.79–2.43)^+^
**A’Sharqiyah** (**North**)								
Baa’d (BD)	150	0.217 (0.181–0.251)	1.55 (0.15)	1.61	3	0.49	2.47 (2.11–2.97)^+^	2.38 (2.01–2.77)^+^
Maqal (ML)	150	0.095 (0.081–0.107)	1.22 (0.20)	1.13	3	0.79	2.27 (1.93–2.81)^+^	1.04 (0.79–1.41)^ns^
Qarr (QR)	150	0.097 (0.085–0.113)	1.19 (0.16)	1.89	3	0.63	1.05 (0.71–1.29)^ns^	1.07 (0.77–1.39)^ns^
Miss (MS)	150	0.137 (0.119–0.156)	1.89 (0.18)	3.11	3	0.21	3.09 (2.77–3.59)^+^	1.51 (1.17–1.96)^+^
**A’Dhahira**								
An Nujayd (AN)	150	0.163 (0.139–0.197)	1.13 (0.16)	3.93	3	0.22	1.45 (1.11–1.87)^+^	1.79 (1.43–2.19)^+^
Al-Hajar (AH)	150	0.166 (0.139–0.196)	1.21 (0.17)	2.97	3	0.37	1.47 (1.09–1.91)^+^	1.82 (1.49–2.27)^+^
**Al-Batinah North**								
Wadi Haibi (WH)	150	0.334 (0.292–0.381)	1.19 (0.17)	1.09	3	0.81	1.89 (1.51–2.47)^+^	3.67 (3.24–3.97)^+^
DEL-SEL	150	0.411 (0.364–0.471)	1.09 (0.15)	2.37	3	0.51	1.82 (1.49–2.27)^+^	4.51 (4.13–4.99)^+^
LAB-SUS (Ref. Strain)	150	0.091 (0.071–0.112)	1.16 (0.19)	1.89	3	0.61	1.04 (0.71–1.33)^ns^	1.00

*n = number of test insects used in bioassay trials, **LC_50_ = median lethal concentration (ppm), ***CI = confidence interval, ****SR = synergism ratio, calculated by dividing LC_50_ value of insecticide of a field strain by the corresponding LC_50_ value of the same insecticide with PBO of the same field strain, *****RR = resistance ratio, calculated by dividing LC_50_ value of a field strain by the corresponding LC_50_ value of the Lab susceptible (Reference) strain, +significantly different from the Lab susceptible (Reference) strain (95% CIs of RR didn’t include 1), and ns = non-significant.

**Table 5:** Effect of PBO on toxicity of fenitrothion in laboratory and field strains of *Ommatissus lybicus* under laboratory conditions.

Field Strain	n^*^	LC_50_^**^(95% CI^***^)	Slope (S.E)	χ^2^	df	P	SR^****^(95% CI)	RR^*****^(95% CI)
**A’Dakhliyah**								
Hidhan (HN)	150	0.026 (0.019–0.034)	1.31 (0.16)	2.58	3	0.51	1.15 (0.83–1.39)^ns^	3.71 (3.21–4.32)^+^
Wadi Qari (WQ)	150	0.058 (0.041–0.079)	1.31 (0.15)	1.89	3	0.63	1.57 (1.21–2.11)^+^	8.29 (7.23–9.01)^+^
Al-Faljayn (AFn)	150	0.030 (0.023–0.038)	1.52 (0.15)	2.96	3	0.47	1.53 (1.16–1.91)^+^	4.29 (4.01–4.81)^+^
Qalat Al-Masalha (QM)	150	0.040 (0.029–0.061)	1.57 (0.15)	5.11	3	0.31	1.58 (1.19–2.04)^+^	5.71 (5.09–6.49)^+^
Al-Faghrah (AFh)	150	0.041 (0.029–0.061)	1.37 (0.17)	0.89	3	0.87	1.71 (1.09–2.67)^+^	5.86 (5.27–6.71)^+^
Al-Jailah (AJ)	150	0.029 (0.018–0.040)	1.51 (0.16)	2.58	3	0.51	1.52 (1.09–1.91)^+^	4.14 (3.51–4.76)^+^
Nafaa (NA)	150	0.018 (0.011–0.029)	1.86 (0.19)	2.68	3	0.51	1.67 (1.27–2.11)+	2.57 (2.19–2.99)^+^
Al-Uaina (AU)	150	0.021 (0.015–0.030)	2.77 (0.25)	4.80	3	0.10	1.38 (1.01–1.76)^+^	3.00 (2.51–3.55)^+^
Al-Elayyah (AE)	150	0.015 (0.010–0.021)	1.79 (0.19)	4.89	3	0.16	1.27 (1.01–1.61)^+^	2.14 (1.81–2.49)^+^
Al-Kharma (AK)	150	0.017 (0.010–0.026)	1.58 (0.15)	4.11	3	0.23	1.47 (1.11–1.91)^+^	2.14 (1.67–2.74)^+^
**A’Sharqiyah** (**North**)								
Baa’d (BD)	150	0.048 (0.037–0.061)	1.27 (0.17)	2.18	3	0.29	1.35 (1.06–1.89)^+^	6.86 (6.33–7.61)^+^
Maqal (ML)	150	0.033 (0.024–0.043)	1.33 (0.16)	0.89	3	0.83	1.30 (1.01–1.77)^+^	4.71 (4.33–5.17)^+^
Qarr (QR)	150	0.038 (0.030–0.051)	1.31 (0.18)	3.21	3	0.19	1.34 (1.03–1.67)^+^	5.43 (5.01–5.99)^+^
Miss (MS)	150	0.035 (0.022–0.049)	1.47 (0.17)	2.19	3	0.29	1.31 (1.07–2.61)^+^	5.00 (4.63–5.49)^+^
**A’Dhahira**								
An Nujayd (AN)	150	0.019 (0.012–0.029)	1.87 (0.19)	2.89	3	0.37	1.32 (1.02–1.69)^+^	2.71 (2.44–3.09)^+^
Al-Hajar (AH)	150	0.014 (0.009–0.020)	1.61 (0.15)	2.25	3	0.28	1.36 (1.08–1.69)^+^	2.00 (1.61–2.49)^+^
**Al-Batinah North**								
Wadi Haibi (WH)	150	0.030 (0.021–0.040)	1.76 (0.17)	3.98	3	0.13	1.30 (1.01–1.67)^+^	4.51 (4.11–4.87)^+^
DEL-SEL	150	0.018 (0.013–0.027)	1.89 (0.19)	3.80	3	0.11	1.39 (1.04–1.81)^+^	2.57 (2.18–3.01)^+^
LAB-SUS (Ref. Strain)	150	0.007 (0.005–0.011)	1.13 (0.17)	2.79	3	0.39	1.14 (0.83–1.43)^ns^	1.00

*n = number of test insects used in bioassay trials, **LC_50_ = median lethal concentration (ppm), ***CI = confidence interval, ****SR = synergism ratio, calculated by dividing LC_50_ value of insecticide of a field strain by the corresponding LC_50_ value of the same insecticide with PBO of the same field strain, *****RR = resistance ratio, calculated by dividing LC_50_ value of a field strain by the corresponding LC_50_ value of the Lab susceptible (Reference) strain, +significantly different from the Lab susceptible (Reference) strain (95% CIs of RR didn’t include 1), and ns = non-significant.

## Discussion

Keeping in view the economic significance of dubas bug, the Ministry of Agriculture and Fisheries, Oman has made efforts for its management in date palm and evaluated several pesticides (OPs and Pyrethroids) by aerial and ground applications. About 400 tons of chemicals costing 9.0 million omani riyals were sprayed during 1993 and 2006^13^. Aerial spraying of insecticides in spring and ground application in winter is carried out every year to control this notorious pest. Despite of extensive application of insecticides, severe infestation of date palms is reported with the pest every year. The development of insecticide resistance in the field strains of dubas bug receiving heavy pesticide regimes can be one of the reasons for incomplete or unsuccessful control^28^. Karimi, *et al*.^29^ suggested that the rapid development of resistance in dubas bug field strains against several insecticides can be closely related to the endosymbiotic bacteria. No documentation of resistance in dubas bug has yet been reported from the studied localities. Furthermore, the study was planned to determine the resistance levels in the field strains of dubas bug against frequently used insecticides (deltamethrin and fenitrothion) to provide base line information and mitigate the risk of resistance development in insect.

The results revealed that out of seventeen field strains tested from various locations in Oman, no field strain possessed high or moderate levels of resistance against deltamethrin. However, one strain (WQ) from A’Dakhliyah governorate exhibited an intermediate level of resistance against fenitrothion. The susceptibility of eleven field strains against deltamethrin (RR < 3-fold) suggests it as suitable choice for future application in DB management program. Fenitrothion proved to be more toxic in our experiments based on LC_50_ comparison, but only two field strains (AE and AH) were found to be susceptible against fenitrothion. Minor to intermediate resistance levels were observed in the remaining fifteen field strains against fenitrothion. The development of minor to intermediate resistance in most of the tested field strains against fenitrothion could be due to heavy spraying of fenitrothion alone and in mixtures through aerial and ground applications. To the best of author’s knowledge, no reports are available documenting the resistance development in dubas bug.

A number of insecticides including chemicals from organophosphate, pyrethroid, neonicotinoid groups and botanicals have been tested for their efficiencies/toxicities against dubas bug under field and laboratory conditions^20,24^. Cypermethrin, deltamethrin, diazinon and fenitrothion were found more toxic and reported to cause high mortality of dubas bug under laboratory and field conditions^25,26^. Bifenthrin and cypermethrin were also reported as more efficient insecticides for dubas bug population reduction up to 35 days of spray in date palm plantations^24^. Insecticide endotherapy (injecting in tree trunk) with thiamethoxam and spraying the foliage with spinetoram were reported very effective against dubas bug^20,27^.

In the present study, a lab strain was re-selected with deltamethrin up to seven generations under laboratory conditions to study the mechanism of resistance against deltamethrin and cross resistance against fenitrothion. The results of the experiments explained the development of rapid but low-level resistance against deltamethrin with an increase in RR value (2.96 to 8.05-folds) with repeated exposures to the insecticide up to seven generations. However, the resistance level was decreased (RR declined from 8.05 to 7.86-fold) with a non-significant difference when 10^th^ generation was exposed to deltamethrin after a gap of exposure for two generations (Gen-8 and 9). These results suggest that spraying with alternate chemicals or no spraying with deltamethrin can delay the development of resistance in dubas bug. Instability and reversion of resistance has been reported in many cases when the selected insects were reared without insecticide exposure for a number of generations^34,38,39^. Further studies are required to confirm the reversion of resistance in dubas bug. Screening of cross resistance potential of field strains against alternate chemicals can also help in mitigating resistance development under selection pressure. In the present studies, fenitrothion was tested against the 10^th^ generation of deltamethrin-selected strain and minor resistance level (3.13-fold RR) was observed suggesting the rotation of the two insecticides in the pest management program.

The Piperonyl butoxide, a recognized oxidase-inhibitor synergistic compound has also shown inhibition of esterases associated with insecticide detoxification^40^ and enhancing the insecticide efficacy in many pests of agricultural importance^41–45^. In the present studies, insecticides (deltamethrin and fenitrothion) were tested along with synergist (PBO) to see the synergistic effects on their toxicities as well as oxidase and esterase-based mechanism of resistance. The results revealed significant reduction in the LC_50_ values of the two insecticides when these were applied in the presence of PBO and suggested the possibility of oxidase or esterase-based resistance in DB. However, synergistic effect of PBO was not observed with the two insecticides against the LAB-SUS strain. Similarly, least synergistic effect of PBO with deltamethrin and fenitrothion was observed in QR strain from A’Sharqiyah (North) and in HN strain from A’Dakhliyah, respectively. In experiments where deltamethrin was applied along with PBO to the field and deltamethrin-selected (DEL-SEL) strains, resistance was reduced from 7.86-fold to 4.55-fold. Minor resistance in WH (RR = 3.67-fold) and QM (RR = 3.60-fold) with SR values of 1.89 and 2.31-fold, respectively was determined against deltamethrin. However, all the remaining field strains proved to be susceptible when treated with deltamethrin in the presence of PBO. These results suggest that application of insecticides in the presence of synergist PBO can help in mitigating the resistance development in the pest. Significant reduction of resistance in different field collected strains of DB was also observed when fenitrothion was used along with the PBO. The resistance status of WQ from A’Dakhliyah governorate was changed from intermediate level (11.38-fold) to low level (8.29-fold) against fenitrothion with PBO. Out of eight strains showing low level of resistance against fenitrothion, four strains (AFn, AJ, ML and MS) exhibited minor level of resistance when fenitrothion was applied in the presence of PBO. Increase in the toxicity of certain insecticides due to PBO has been reported against several pests of agricultural significance^40–45^. Lack of information on the resistance development in dubas bug suggests further studies to determine the exact mechanism of resistance based on biochemical and molecular investigations.

Climatic suitability of Oman can serve conducive for the severe infestations of dubas bug in future^16^ and the resistance development can contribute to its management challenges for the plant protection department. Fortunately, high resistance has not been observed in any field strain tested against the two insecticides. However, the continuous spraying with same insecticides may result in quick development of resistance due to enhanced levels of esterase or oxidases. Higher toxicity of the pesticides in the presence of PBO encourages the use of synergists along with pesticides. Furthermore, the pesticide application in rotation along with other integrated pest management practices can also help in mitigating the resistance development in *Ommatissus lybicus*.

## Materials and Methods

### Insects

**Figure 1:**
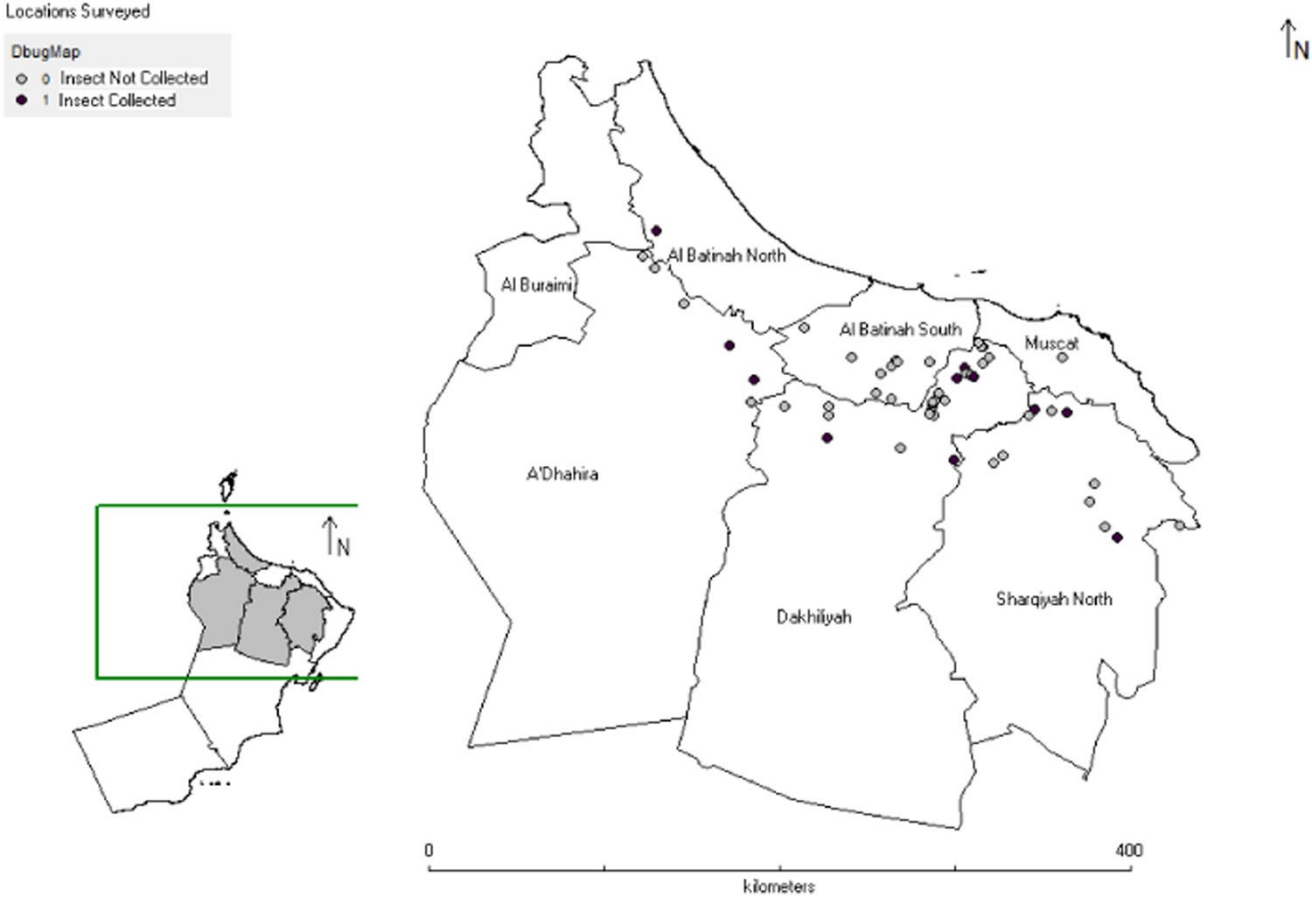
Locations surveyed for insect collection.

**Figure 2:**
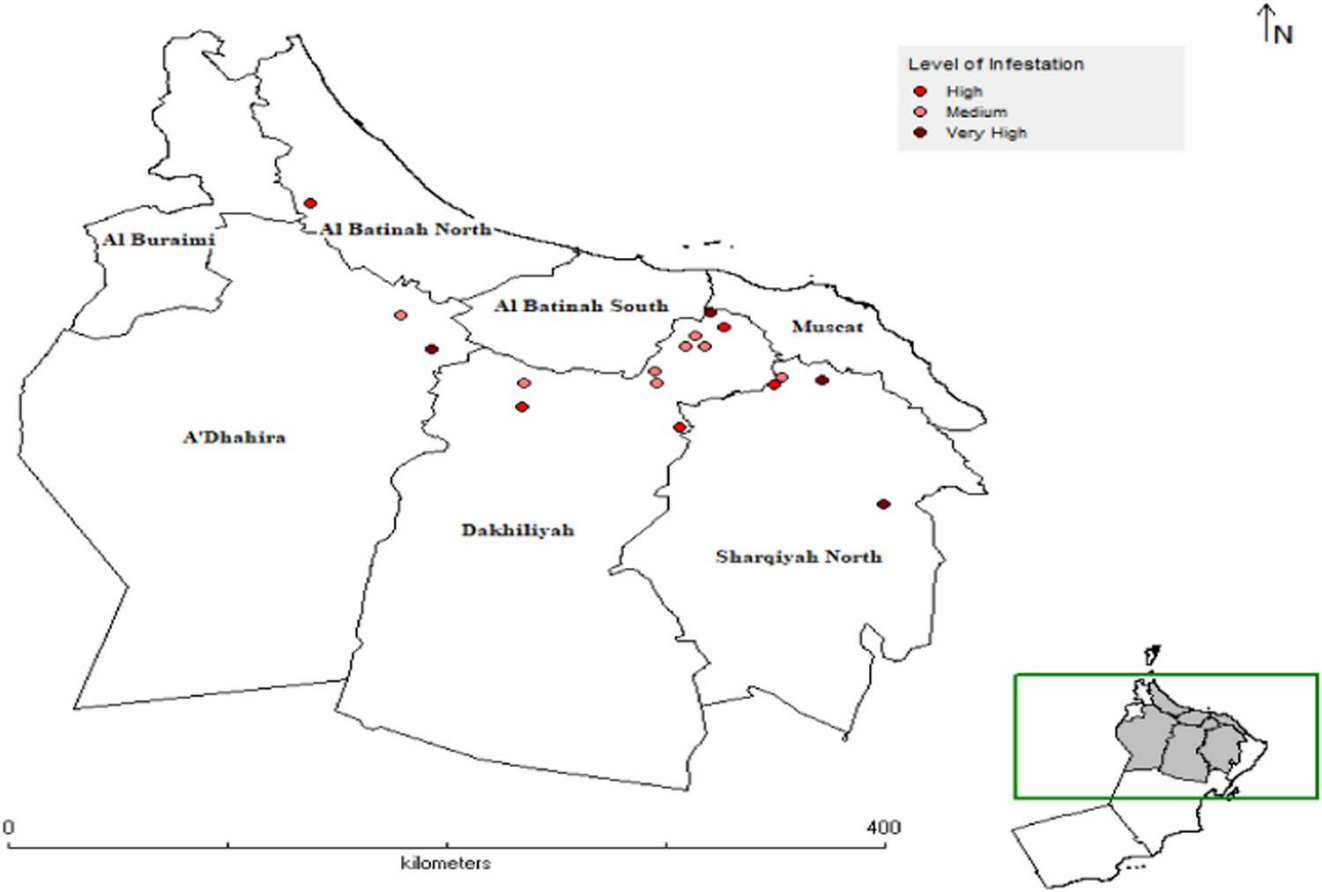
Sites for insect collection and infestation levels.

Extensive surveys of almost fifty locations from different governorates of Oman were carried out during the year 2017 and 2018 to collect the field populations of dubas bug, however, seventeen field strains were collected based on pest infestation and target stage (Figs 1 and 2; Table 6). The insects were maintained on their natural host (Potted date palm off-shoots) in mesh cages (height = 80 cm, width = 60 cm and depth = 50 cm) and glass cages (height = 150 cm, width = 100 cm and depth = 80 cm) under the laboratory conditions 27 ± 2 °C, 70 ± 5% RH and 12 L:12D photoperiod. A strain was collected from unsprayed area in 2013 and maintained under the same lab conditions as explained above. This lab strain was never exposed to any insecticide and was used as lab-susceptible reference strain (LAB-SUS). A population from the lab strain was selected consecutively by performing bioassay experiments with deltamethrin till seven generations and its 10th generation was used as deltamethrin-selected strain (DEL-SEL). The survivors in each bioassay were reared as deltamethrin-selected strain at the same conditions as explained above and was used in bioassay experiments for comparison with field strains.

**Table 6:** List of locations and insect infestation.

Location	Date of insect Collection	Coordinates		Insect Stage	Infest. Level
		Lat.	Long.		
**A’Dakhliyah**					
Hidhan (HN)	23.04.2017	23.48018	58.08596	Nymph	Very High
Wadi Qari (WQ)	26.04.2017	23.16402	57.85080	Nymph	Medium
Al-Faljayn (AFn)	06.11.2017	23.09832	57.85911	Nymph/Adult	Medium
Qalat Al-Masalha (QM)	07.11.2017	23.09843	57.31124	Nymph/Adult	Medium
Al-Faghrah (AFh)	04.04.2018	23.29677	57.97801	Nymph	Medium
Al-Jailah (AJ)	04.04.2018	23.35111	58.01806	Nymph	Medium
Nafaa (NA)	09.04.2018	23.40571	58.14156	Nymph	High
Al-Uaina (AU)	09.04.2018	23.29778	58.05972	Nymph	Medium
Al-Elayyah (AE)	10.04.2018	22.97426	57.30080	Nymph/Adult	High
Al-Kharma (AK)	10.04.2018	22.85743	57.95805	Nymph/Adult	High
**A’Sharqiyah** (**North**)					
Baa’d (BD)	12.11.2017	23.09430	58.34899	Nymph/Adult	High
Maqal (ML)	12.04.2018	22.44620	58.80078	Nymph/Adult	Very High
Qarr (QR)	09.04.2018	23.11570	58.54346	Nymph/Adult	Very High
Miss (MS)	09.04.2018	23.12692	58.37800	Nymph	Medium
**A’Dhahira**					
An Nujayd (AN)	11.04.2018	23.46539	56.79447	Nymph/Adult	Medium
Al-Hajar (AH)	11.04.2018	23.28242	56.92563	Nymph	Very High
**Al-Batinah North**					
Wadi Haibi (WH)	16.04.2018	24.07614	56.42078	Nymph	High

### Insecticides

The two insecticides [Decis 2.5EC (deltamethrin; Bayer Crop Sciences, Monheim am Rhein, Germany) and Sumithion 50EC (fenitrothion; Sumitomo Chemical, Tokyo, Japan)] and Piperonyl butoxide [PBO(purity >90%); Sigma-Aldrich Chemie GmbH (Merck), Taufkirchen, Germany], were provided by the Department of Plant Protection, Directorate General of Agricultural and Livestock Research, Ministry of Agriculture and Fisheries, Oman and were used for bioassay experiments.

### Bioassays and selection experiments

The bioassay experiments were performed by following the methodology explained by Dang *et al*.^33^. The test insecticide solutions with a range of concentrations demonstrating ˃0% and ˂100% mortality (estimated through pre-bioassay tests), were prepared in acetone. The insecticide concentrations ranging from 0.062–1.00 ppm and 0.015 to 0.250 ppm were used for deltamethrin and fenitrothion, respectively. The volume measuring 1 ml of the acetone diluted insecticide was added to a glass petri dish (90 cm diameter × 18 mm height). The petri dish was rotated gently to ensure complete and even application of insecticide. The treated petri dishes were then placed under the fume hood until 24 hours for complete evaporation of acetone and uniform application of insecticide residues on the glass surface. The control glass petri dishes were prepared by applying the same volume of acetone (without insecticide). A counted number of healthy homogeneous insects (3^rd^ instar) of each strain were released in the petri plates treated with the insecticides. The observations for the dead/alive insects were noted after 48 hours of treatment application. The insects were considered dead with no signs of movement (appendages) by touching with the camel hair brush. Three replicates were used for all insecticide treatments as well as control. The synergism bioassays were performed by applying the PBO before the application of insecticide dilutions in the petri dishes^34^.

The deltamethrin-selected strain was also used in bioassay experiments for resistance comparison with lab-susceptible and field collected strains. A population from the existing lab strain was selected consecutively by exposing to deltamethrin dilutions (70% mortality) till seven generations (G-0 to G-7) and its 10th generation was used as deltamethrin-selected strain (DEL-SEL). The generations (G-8 to G-9) were not exposed to the insecticide. The survivors in each bioassay were reared as deltamethrin-selected strain at the same conditions as explained above and was used in bioassay experiments for comparison with field strains^34^.

### Data analyses

The median lethal values of the insecticide concentrations (LC_50_) and confidence intervals (CIs) were calculated by the Probit analysis of the mortality scores in the treatments using Polo Plus software version 1.0 (LeOra software LLC). Significant differences among the LC_50_ values were only considered with non-overlapping CIs in the respective experiment. The field strains treated with the two insecticides and displaying slope values >0.90, indicate low phenotypic variation among the tested individuals of *O*. *lybicus*^35^. Resistance ratios (RRs) for the field and DEL-SEL strains were calculated by dividing the LC_50_ of the respective strain by that of the lab-susceptible (LAB-SUS) strain. The resistance levels were categorized by adopting the criterion explained by Jin *et al*.^36^, susceptible (RR less than 3-fold), minor resistance (RR = 3–5-fold), low resistance (RR = 5–10-fold), intermediate resistance (RR = 10–40-fold), high resistance (RR = 40–16-fold), and extremely high resistance (RR greater than 160-fold). Confidence levels (95% CIs) of resistance ratios (RR) at LC_50_ levels were calculated to see the significant difference between any two (Ref. vs field strain) LC_50_ values. The resistance ratios (RR values) were considered significantly different if the 95% CI does not include 1^37^. The synergism ratios (SRs) were calculated by dividing the LC_50_ value of insecticide of a strain with synergist (PBO) by the LC_50_ value of the same strain with out synergist. The significant effects by the synergistic chemical were assessed by calculating the 95% CIs of the synergism ratio (SR) at LC_50_ level and were used as stated above.

### Ethical statement

The article deals with an insect species *Ommatissus lybicus*, belonging to phylum arthropoda and reports the results obtained from standardized experiments. The bioassay procedures were approved by the Department of Plant Protection, Directorate General of Agriculture, Ministry of Agriculture and Fisheries, Oman. No experiments were performed with human participants by any of the authors.

## Data Availability

Statistically analyzed data are presented in the manuscript, however, the data and observations noted in experiments are submitted to the Department of Plant Protection, Directorate General of Agriculture and Livestock Research, Ministry of Agriculture and Fisheries, Oman and The Research Council (TRC), Oman. The data can be only available upon the consent of the concerned authorities.
